# The Interaction of Target and Masker Speech in Competing Speech Perception

**DOI:** 10.3390/brainsci15080834

**Published:** 2025-08-04

**Authors:** Sheyenne Fishero, Joan A. Sereno, Allard Jongman

**Affiliations:** Linguistics Department, The University of Kansas, Lawrence, KS 66045, USA; snfishero@gmail.com (S.F.); sereno@ku.edu (J.A.S.)

**Keywords:** speech segregation, speech perception, target speech, masker speech, background noise, f0, speaking rate, rhythm class

## Abstract

**Background/Objectives**: Speech perception typically takes place against a background of other speech or noise. The present study investigates the effectiveness of segregating speech streams within a competing speech signal, examining whether cues such as pitch, which typically denote a difference in talker, behave in the same way as cues such as speaking rate, which typically do not denote the presence of a new talker. **Methods**: Native English speakers listened to English target speech within English two-talker babble of a similar or different pitch and/or a similar or different speaking rate to identify whether mismatched properties between target speech and masker babble improve speech segregation. Additionally, Dutch and French masker babble was tested to identify whether an unknown language masker improves speech segregation capacity and whether the rhythm patterns of the unknown language modulate the improvement. **Results**: Results indicated that a difference in pitch or speaking rate between target and masker improved speech segregation, but when both pitch and speaking rate differed, only a difference in pitch improved speech segregation. Results also indicated improved speech segregation for an unknown language masker, with little to no role of rhythm pattern of the unknown language. **Conclusions**: This study increases the understanding of speech perception in a noisy ecologically valid context and suggests that there is a link between a cue’s potential to denote a new speaker and its ability to aid in speech segregation during competing speech perception.

## 1. Introduction

In daily life, speech perception typically happens in noisy environments with other background sounds. These background sounds can cause physical interference with the speech signal that results in greater difficulty in perceiving speech. This physical acoustic interference between environmental noises and target speech is called energetic masking [1]. When speech is masked by other speech, there is additional perceptual interference that occurs, called informational masking [1,2]. Due to this additional perceptual interference, the perception of speech in competing speech is typically more difficult than perceiving speech in non-speech noise [3,4,5]. Because speech perception does not typically occur in a quiet environment, and because competing speech typically does not share all acoustic properties with target speech, understanding the acoustic variables that influence competing speech perception and how they interact is critically important in understanding the nature of speech perception in an ecologically valid context. In the present study, the interaction of f0 and speaking rate is examined.

A number of variables have been examined that influence competing speech perception. These variables include the characteristics of the target speech that listeners are instructed to pay attention to and the characteristics of the masker speech, which is typically multi-talker babble, superimposed over the target speech to experimentally create an environment of competing speech perception.

One reason why the perception of target speech in masker speech is particularly difficult compared to speech-in-noise perception stems from the lexical competition that occurs due to masker speech. Brungart and Simpson [6] observed lexical competition during competing speech perception using Coordinate Response Measure (CRM) stimuli [7] that include combinations of numbers and colors presented on a grid on a monitor. In this task, a number and color combination is auditorily presented within another number and color combination masker phrase, and participants are tasked with selecting the proper combination of number and color from the set of possible options on the screen. Results on this task demonstrated that when participants made mistakes, the mistake typically matched the content of the masker speech, meaning the participant had trouble disambiguating between the target and masker, rather than trouble with perceiving the target speech signal. Additionally, removal of the response that corresponded with the masker speech signal from the set of possible response options on the screen led to a dramatic improvement in performance on the task, indicating again that most errors on the task were due to misidentifying the masker as the target. These results offer evidence that lexical competition between target and masker speech influences performance on competing speech perception tasks.

Brouwer and Bradlow [8] used eye-tracking to study lexical competition during competing speech perception. Their experiment involved disyllabic nouns where target and competitor words matched in either onset or offset. Participants viewed displays containing the target (e.g., ‘candle’), onset competitor (e.g., ‘candy’), rhyme competitor (e.g., ‘sandal’), and a distractor (e.g., ‘lemon’) while hearing a target word and a competing word spoken simultaneously by different female speakers. The target was 2 dB louder than the masker. Results showed greater visual fixations on onset-matching competitors compared to distractors, regardless of the masker type. Additionally, rhyme-matching competitors attracted more fixations than distractors and were preferred over onset-matching competitors when the masker speech matched the rhyme. This indicates that background speech can cause lexical competition with target speech, and the onset competitor advantage that is typically expected in visual world eye-tracking research [9] can be reduced when the rhyme competitor is played as masker background speech. These results demonstrate that lexical competition from background speech influences lexical processing of a target speech signal.

Additionally, lexical properties of the masker speech modulate its effectiveness as a masker. For example, previous research has found that meaningful masker speech masks target speech more effectively (more interference) than nonmeaningful masker speech [10,11]. Furthermore, masker speech containing high-frequency words has been found to mask target speech also more effectively than masker speech containing low-frequency words [12].

While previous research has identified that there is a clear role of lexical competition from the masker speech signal during competing speech perception, much less is understood about what types of acoustic differences/similarities between target and masker influence competing speech perception.

Some previous research seems to suggest that increased acoustic similarity between target and masker speech is a major detriment to target speech perception. One acoustic cue that has been well-studied in the competing speech perception domain is f0. Previous research has found that when target and masker speech mismatch in speaker sex, which typically corresponds to differences in f0 and formant frequencies, the masker is less effective at masking the target speech signal [13,14,15]. This phenomenon was demonstrated in Brungart [13] through the use of the Coordinate Response Measure (CRM) task using target speech played within single-talker masker speech that was either produced by the same talker as the target speech, from a different talker of the same sex as the target speaker, or from a different talker of a different sex as the target speaker. Participants were more accurate at segregating the target speech signal from the masker speech signal when the masker was of a different sex from the target compared to the same sex, and participants were more accurate perceiving the target speech signal when the masker speaker differed from the target speaker, even if the masker speaker was of the same sex as the target speaker. When using multi-talker (3- and 4-talker) masker babble, similar results were found, with target speech perception best when target and masker speech differed in sex and worst when target and masker speech were produced by the same talker [14]. These studies indicate that f0 differences between target and masker speech can greatly improve competing speech segregation and offer release from masking.

This phenomenon of mismatches in sex between target and masker offering release from masking also occurs when artificially changing f0 to approximate sex differences [16]. In this study, speech from the same talker was used as the target and masker speech. The masker speech was composed of only single-talker speech, not multi-talker babble. f0 differences between target and masker were artificially manipulated to fall within 0 and 12 semitones. Results indicated gradual improvement in target sentence perception as the difference in f0 between target and masker increased. This improvement, even at the largest f0 difference (12 semitones), was not as significant as the improvement in the perception of target speech masked by a single masker talker of a different sex than the target.

Similarly, previous research using different sentential stimuli has also found that masker speech in a different pitch range from target speech is less effective at masking compared to speech in similar pitch ranges, and greater differences between target and masker pitch lead to less and less effective masking [17]. In this study, the f0 of syntactically correct semantically anomalous sentences was artificially manipulated to 100, 103, 106, 109, 120, and 200 Hz for the target speech sentences. The masker speech was a continuous speech stream manipulated to an f0 of 100 Hz. Accuracy scores on a sentence repetition task indicated that error rates generally reduced as the difference in f0 between target and masker increased. When using natural productions of f0 to create distinct pitch ranges instead of artificially manipulating the pitch range (the low f0 stimuli comprised a male’s normal pitch utterances, while the high f0 stimuli comprised the same male speaker’s utterances when he attempted to imitate a female speaker’s pitch), results again indicated that error rates were lowest when target and masker differed in f0 pitch range compared to when they were from a similar pitch range. Additionally, f0 contour differences (either flat, normal, or exaggerated speaking style) between target and masker speech decrease the effectiveness of masker speech at masking the target [18].

These previous studies indicate that differences in f0 properties between target and masker speech robustly aid in the segregation of a competing speech signal, regardless of whether the differences arise from natural productions or artificial manipulation.

While the role of f0 has been studied in competing speech perception, little research has identified the role of speaking rate in masker effectiveness. Calandruccio et al. [19] indirectly investigated the role of speaking rate in competing speech perception by testing the perception of English clear and conversational target speech within English and Croatian clear and conversational masker speech. Due to the slower speaking rate of clear speech compared to conversational speech, this study partially tests the role of speaking rate, although other acoustic differences exist between clear and conversational speech beyond just speaking rate (such as differences in vowel reduction, consistency in release of stop bursts, and RMS intensity for obstruent sounds [20]), meaning any findings of the study cannot be exclusively attributed to speaking rate. Participants in the experiment listened to female target speech spoken in either conversational or clear speaking style within female two-talker masker babble composed of either English clear speech, English conversational speech, Croatian clear speech, or Croatian conversational speech. Participants were not familiar with Croatian. Accuracy scores in a sentence repetition task in which participants were supposed to repeat aloud what the target speaker just said indicated that while clear target speech has an expected perceptual advantage over conversational target speech, masker speaking style (clear versus conversational) did not influence masker effectiveness. While these data suggest slower speaking rates in clear speech may improve intelligibility of target speech during competing speech perception, the clear speech benefit may not only be attributed to speaking rate given other potential acoustic differences between clear and conversational speech.

Speech rhythm has also been shown to play a role in competing speech perception. Using artificially manipulated speech rhythm irregularities in target and masker speech, McAuley [21,22] found that masker speech with an irregular rhythm led to improved target speech perception. However, when f0 differences were available as a cue, speech rhythm irregularities no longer mattered. Within these studies, the rhythm irregularities in the stimuli were created by speeding up and slowing down the speech in a sinusoidal pattern in order to create a disruption from the natural sentence timing.

Studies have also been conducted on speech rhythm differences in target and masker speech by studying languages with differing rhythm classes [23,24]. Rhythm classes are a means of categorizing languages based on rhythmic properties, but the exact criteria that are used when classifying languages have been highly debated in the literature [25,26,27,28]. Two of the three main rhythm classes that have been identified are stress-timed languages for which stress is said to occur at equal intervals and syllable-timed languages for which syllables tend to occur at equal intervals. Studies have found that languages that allow for complex syllables or vowel reduction are typically stress-timed languages, and languages that allow for only simpler syllable structures and no vowel reduction are typically syllable-timed languages [29]. Acoustic studies (e.g., [28]) have indicated that rhythm classes can sometimes be identified through the duration of intervals between vowels and between consonants. However, Arvaniti [25,26] claims that cross-linguistic differences between rhythm classes are not reliably supported based on acoustic measures and argues instead that rhythm research should focus on prominence and grouping measures of languages as opposed to the more traditionally defined stress-timed and syllable-timed type distinctions.

For the languages of the current study (English, Dutch, and French), previous research largely points to English and Dutch sharing properties traditionally corresponding to stress-timed languages, while French has properties traditionally corresponding to syllable-timed languages. Ramus et al. [28] found that when plotting the proportion of vocalic intervals and the average standard deviation of consonantal intervals, English and Dutch clustered together, while French was plotted in a different cluster farther away, suggesting that English and Dutch belong to a different rhythm class from French. Similarly, when studying prominence features across languages, Dutch and English share similar prosodic structures [30] and have lexical stress [31,32], while French uses prosody differently when cueing focus [33] and has phrasal prominence instead of lexical stress [34]. Overall, given these characteristics of English, Dutch, and French rhythm and prominence, English and Dutch share many similarities that are distinct from French. For the purposes of the present study, the terms stress-timed (for English and Dutch) and syllable-timed (for French) will be used to represent these differences between languages, although it should be noted that these terms are not agreed upon by all researchers.

Reel and Hicks [24] compared the perception of English (a stress-timed language) target speech within varying masker languages that either match or mismatch English in rhythm class. When comparing the effectiveness of English, German, French, Spanish, and Japanese maskers at masking English target speech, they found that French and Spanish, which do not share a rhythm class with English, were less effective at masking compared to German and English, which do share a rhythm class with the English target. However, they additionally found that Japanese, which does not share a rhythm class with English, was equally effective at masking English as German. This could potentially be due to Japanese sharing some properties with stress-timed languages such as English and German, but it could potentially point to a more complex relationship between masker effectiveness and language properties than simply rhythm class.

Previous research robustly finds that when the masker speech is in a language unknown to the listener, it is less effective at masking target speech than masker speech in a known language [8,23,24,35,36,37,38,39,40,41,42]. Additionally, Calandruccio et al. [39] showed that unknown languages that are similar to the target (Dutch masker for English target) mask more effectively than dissimilar languages (Mandarin masker for English target). Results indicated that English maskers were most effective at masking English target speech, with Dutch the next most effective and Mandarin the least effective.

A potential confound in the study by Calandruccio et al. [39] was that the Mandarin masker babble had less high-frequency energy than the English and Dutch maskers. A follow-up study was conducted with new maskers consisting of noise files that were spectrally matched to the English, Dutch, and Mandarin babble from the initial experiment. This allowed for the examination of spectral differences between English, Dutch, and Mandarin while removing any low-frequency temporal modulation differences that may have been present in the initial experiment. Additionally, another set of maskers was created with white noise that matched the low-frequency temporal modulations from the English, Dutch, and Mandarin babble maskers from the original experiment, which allowed for the study of the impact of temporal information on masker effectiveness. The use of white noise meant the spectral differences that typically appear for English, Dutch, and Mandarin were not present, so any differences found in masker effectiveness could be attributed to the low-frequency temporal modulations. Results indicated that the Mandarin masker babble’s spectral energy was less effective at masking the target compared to the English and Dutch maskers, meaning the results from the initial experiment do not fully represent differences in linguistic and phonetic properties of English, Dutch, and Mandarin. The fact that English and Dutch maskers were equally effective for the spectrally matched noise conditions, suggests that something beyond just spectral differences drove the results from the initial experiment where English was a more effective masker than Dutch. The findings suggest that the benefit of English over Dutch over Mandarin in masker effectiveness could result from rhythm class differences between the languages.

Overall, these experimental results hint at a potential role of rhythm class in masker effectiveness. The findings indicate that unknown language maskers are less effective than a known language masker and differ from each other in terms of effectiveness.

Target and masker mismatch in terms of dialect, such as General American English masking Southern American English, also leads to less effective masking of target speech [43]. Additionally, a foreign-accented masker is less effective in masking natively accented target speech [44]. Finally, when comparing target speech perception within masker speech of the same language as the target (English) or a different language (Greek), which, crucially, is also known by the listeners, it has also been found that the target language masker is the more effective masker [23]. This study tested monolingual English and Greek–English bilinguals’ ability to segregate English target speech from either English or Greek two-talker masker babble. Results indicated that Greek masker speech was a less effective masker compared to English speech, even for the bilingual listeners who were familiar with both languages. Because the participants understood both the target and masker language in this study, these results cannot be explained by differences in lexical competition between the language match and mismatch conditions. Instead, the authors posited that these differences in performance may result from syllable rate or general rhythm pattern differences between the target and masker speech when spoken in different languages that may modulate masker effectiveness.

The consistent finding of all these studies is that increased similarity between the target and masker hinders the segregation of target speech from masker speech, often called the *target-masker linguistic similarity hypothesis* [38]. The present study further explores the target-masker linguistic similarity hypothesis by systematically examining the influence of both fundamental frequency (f0) and speaking rate differences between target and masker speech. Specifically, it investigates f0 and speaking rate as distinct sources of variability. Prior research on earwitness testimony has highlighted significant differences in how memory biases affect these two acoustic features. When recalling a voice from memory, listeners tend to perceive lower f0 pitches as even lower and higher f0 pitches as even higher than they originally were. However, no such memory bias has been observed for speaking rate differences [45]. Mullennix et al. [45] attributed this to the transient nature of surface speech properties like speaking rate, which can vary significantly within a single speaker and thus provide an unreliable cue for speaker identification. In contrast, f0 exhibits less within-speaker variation, making it a more stable and reliable cue for distinguishing speakers. As a result, f0 is more likely to be stored in memory and subject to perceptual distortions, whereas speaking rate is not. Thus, while f0 serves as a memory cue for speaker identification, speaking rate does not, indicating that they may play fundamentally different roles in speech perception.

Beyond assessing the independent contributions of f0 and speaking rate differences between target and masker speech, this study also examines their combined effect to determine whether variations in both properties influence masker effectiveness differently than changes in just one. Additionally, the research introduces unfamiliar masker speech to investigate whether the absence of attention to fine-grained acoustic differences alters their importance in modulating speech perception during competition. This unfamiliar masker speech includes languages from the same and different rhythm classes as the target language, allowing for an analysis of how rhythm similarities affect speech perception in competing speech conditions.

To gain deeper insight into how multiple cues interact in competing speech perception, this study assesses whether cues not stored in memory for speaker identification, such as speaking rate, function differently from those that are, such as f0. This analysis will focus on their effectiveness in separating target speech from masker speech in a competing speech environment. Furthermore, the interaction between f0 and speaking rate may vary based on whether listeners are familiar with the masker speech and whether it shares a rhythm class with the target speech. Understanding these interactions will enhance our insight into the cognitive processes involved in competing speech perception.

In summary, while prior research has identified various factors affecting the effectiveness of masker speech, little is known about how multiple acoustic cues interact during competing speech perception. The present research comprises five experiments that systematically investigate the role of f0 and speaking rate in modulating target speech perception. The goal is to determine how these cues differentially influence listeners during competing speech perception due to their varying ability to signal a new speaker. Moreover, by incorporating unknown masker speech from different rhythm classes, this study examines whether the effects of speaking rate and f0 differ when listeners do not understand the masker speech and whether rhythm class or speaking rate plays a more significant role in shaping competing speech perception. The findings will contribute to a broader understanding of how multiple acoustic cues interact in speech perception, ultimately informing our knowledge of the factors influencing real-world speech processing in noisy environments.

Five experiments were conducted. Procedures and stimuli are similar across all experiments. They will be described in detail for Experiment 1; subsequently, any differences in procedures as well as specific stimulus information will be introduced for each experiment. Experiment 1 tested whether having a similar f0 range for talker and masker speech will result in more effective masking compared to having a distinct f0 range.

## 2. Experiment 1: The Role of f0

### 2.1. Participants

#### 2.1.1. Speakers

Speakers included three female native English speakers (age range 23–28 years old, mean age 25.67 years old) of a Midwestern dialect of American English, two of whom made up the two-talker masker babble and one of whom was the target speaker. Speaker 1 (age 28) had a mean speaking rate of 4.4 syllables per second (SD = 0.7 syllables/second) and a mean f0 of 197 Hz (SD = 43 Hz). Speaker 2 (age 23) had a mean speaking rate of 4.0 syllables per second (SD = 0.4 syllables/second) and a mean f0 of 187 Hz (SD = 48 Hz). Speaker 3 (age 26) had a mean speaking rate of 5.0 syllables per second (SD = 0.6 syllables/second) and a mean f0 of 246 Hz (SD = 49 Hz). Speaker 1 was selected as the target speaker due to her middle range value for speaking rate and f0. Speakers 2 and 3 served as the masker speakers.

#### 2.1.2. Listeners

Listeners included 20 native English speakers (10 males and 10 females; mean age 34.5 years old). Ten listeners were presented with a low f0 target talker within either low f0 or high f0 two-talker masker babble (half of the trials were low and half were high), and 10 were presented with a high f0 target talker within either low f0 or high f0 two-talker masker babble. Participants were recruited from Prolific (prolific.co) [46] and were paid $10 per hour for their participation. In order to be eligible for recruitment, participants had to live in the United States, be between 18 and 65 years of age, and speak English as their native language.

### 2.2. Materials

Stimuli comprised a subset of the 1965 Revised List of Phonetically Balanced Sentences (Harvard Sentences) [47]. For the 720 total sentences, there are on average 8.31 words per sentence (range 5 to 11 words). Most sentences are declarative, and words are largely one or two syllables long (although some words have more than two syllables, for example, *unfortunately*). Each sentence is composed of five key words. Two examples of these sentences include *Rice is often served in round bowls,* and *These days a chicken leg is a rare dish*. The selection of the subset of stimuli used in the present experiment was arbitrary, although interrogative sentences were avoided.

In order to create target speech within two-talker masker babble, all talkers produced 300 sentences in a quiet room remotely. The set of stimuli was repeated so that each participant recorded a total of two repetitions of each utterance. The first utterance of each stimulus sentence was used unless background noise, lip smacks, or any other imperfection was present, in which case the second repetition of the utterance was used (111/900 = 12.3%). Stimulus sentences were presented to speakers in PowerPoint form with breaks approximately every 50 slides. The initial sentence of the recording session, as well as each initial sentence after each break, was treated as a filler to avoid prosodic list effects.

#### 2.2.1. Masker Babble Creation

For the two speakers whose utterances comprised the masker babble, 165 sentences not used as target speech were selected as the masker stimuli. The intensity of these stimuli was RMS normalized in Praat (v. 6.4.12) [48]. The speech files of each of the two talkers were then concatenated to create one long string of stimulus sentences without any silence between them. At this point, f0 (and speaking rate) could be manipulated.

f0 levels were adjusted for both masker speakers to fall into a high (225 Hz) and a low (175 Hz) f0 range using Praat [48], representing values that fall within the typical range of female speech. The values were intended to fall near the low and high end of the continuum for female speech, at more extreme values than the average f0 value of the speaker selected as the target (197 Hz) and at more extreme values than the average f0 value of all three speakers whose voices were recorded for this experiment (average of 210 Hz). The f0 values selected were played to three phoneticians to ensure they were perceived as natural f0 values for women and that the low value was perceived to have a low pitch and the high value perceived to have a high pitch. This resulted in two sets of babble files per masker babble speaker (one for high f0 stimuli and one for low f0 stimuli).

In order to ensure any masking effects are not due to speaking rate, the speaking rate of all utterances was also adjusted to a neutral rate of 4.5 syllables per second using the overlap-add feature of Praat [48]. This feature uses the Time-Domain Pitch-Synchronous Overlap-and-Add (TD-PSOLA) method to manipulate the speaking rate in a more naturalistic way, not just a uniform way for all speech sounds [49]. The 4.5 syllables per second value was chosen because it is equidistant between the fast and slow rates chosen for Experiment 2, it represents the average speaking rates of the speakers’ natural productions (4.5 syllables per second), and it was perceived as a natural conversational speaking rate by three phoneticians.

Next, the concatenated and manipulated files were combined for the two babble speakers in Praat so that the high f0 stimuli were combined together for both speakers and the low f0 stimuli were combined together for both speakers to create the masker babble. The files for each speaker were not exactly the same duration, so the excess duration of the longer file was removed to ensure there are always two speech streams within the masker babble. At this point, the resulting stereo file containing the two speech files was converted to mono in Praat. The masker babble files for all five experiments of this study were also all normalized for long-term average speech spectrum (LTASS) to avoid differences in con masking across conditions (The script used for this was provided by Dr. Susanne Brouwer. It was created by Daniel, McCloy and modified by Chun Chan; it was loosely based on a script by Veenker, Van Delft, and Quené. While our use of combining mono-channel speech files replicates the methodology used in the vast majority of speech-in-speech perception studies, a reviewer pointed out that this may make it more difficult to evaluate the nature of energetic masking, as compared to dichotic stimulus presentation (cf. [50,51]). Our use of LTAS normalization does mitigate this concern but does not allow us to address the extent to which specific items are subject to energetic masking).

#### 2.2.2. Target Sentence Creation

The target speech files included 100 stimulus sentences from Speaker 1 who was not one of the masker babble speakers. None of the 100 target stimuli was included within the masker babble. The files were once again individually manipulated for f0 and speaking rate in the same way as the masker babble files using the overlap-add function of Praat. Each stimulus sentence was created with a high f0 of 225 Hz and a low f0 of 175 Hz. All stimuli were also manipulated to a neutral speaking rate of 4.5 syllables per second.

In the experiment, target f0 was a between-subjects variable. This was done to ensure each participant only heard the same target f0 value throughout the experiment to avoid confusion about which voice was the target voice. As a result, all stimulus sentences were required to be created with both a high f0 and low f0 value since half of the participants would hear the 100 stimuli with a high f0 value, and the other half would hear the 100 stimuli with a low f0 value.

#### 2.2.3. Combining Masker Babble and Target Sentences

To create target speech within masker babble, the target files and masker babble files were combined. All combinations of target and masker f0 were created so that there were 50 stimuli with a high f0 target and high f0 masker, 50 stimuli with a high f0 target and low f0 masker, 50 stimuli with a low f0 target and a low f0 masker, and 50 stimuli with a low f0 target and a high f0 masker.

In order to identify where in the masker babble file to begin excising babble to combine with the target speech, a random integer was generated to represent that start time of the masker babble for each experimental trial token. At this timepoint, a portion of babble was selected from the masker babble file beginning at the randomly generated start time and ending so that the duration of the masker babble was 1 s longer than each target file’s duration. This allowed for an additional 500 ms of masker babble at the beginning and ending of each embedded target stimulus sentence. The masker babble file selected was RMS-normalized to an intensity of 66 dB, and the target file was RMS-normalized to an intensity of 67 dB to create an SNR of +1 dB. This SNR was selected as a result of pilot studies indicating that less favorable SNRs such as 0 dB, −1 dB, and −3 dB all produced very poor accuracy scores. In order to ensure participants would not find the task too difficult to complete, an SNR of +1 dB was selected.

Prior to combining the target and masker stimuli, 500 ms of silence was added to the beginning and end of each target file to ensure there would be a 500 ms cushion at the beginning and end of each experimental file that only contained masker babble. At this point, the target and masker files were combined in Praat. The resulting file was converted from stereo to mono. Finally, the first 100 ms and last 100 ms of babble were tapered using the Fade function within the Praat Vocal Toolkit [52] to ensure the amplitude was gradually increased and gradually decreased at the beginning and end of each experimental token, respectively, thus avoiding any rapid transitions.

A subset of the 200 stimuli was created with a more favorable SNR to serve as familiarization stimuli. These stimuli were played to participants prior to the experimental stimuli to acclimate them to the target speaker. Each participant heard 20 familiarization stimuli for this experiment so that they knew who the target speaker was. Due to the between-subjects design of the experiment, 20 stimuli with a high f0 and 20 stimuli with a low f0 value had to be created, thus resulting in a total of 40 stimuli for the familiarization phase. For the familiarization phase stimuli, the masker babble files were RMS normalized to an intensity of 52 dB to create an SNR of +15 dB. Therefore, of the 200 stimuli, 160 had an SNR of +1 dB and 40 an SNR of +15 dB).

Each participant only heard the target as always high f0 or always low f0 within both high f0 and low f0 two-talker masker babble. The first 20 stimuli that each participant heard were those with an SNR of +15 dB (familiarization), and the subsequent 80 stimuli were those with an SNR of +1 dB (experimental). The order of presentation of stimuli within the familiarization phase and the experimental phase was randomized across participants.

### 2.3. Procedure

The experiment was conducted remotely online using Gorilla Experiment Builder [53]. Participants first completed the consent process and then were asked a set of eligibility questions. Participants under 18 and over 65, as well as non-native English-speaking participants, were excluded from the experiment. Participants then completed a headphone task [54] to ensure they wore headphones during the experiment. This task played three audio signals, one of which was 180° out of phase, and participants were instructed to select the quietest tone. If participants were not wearing headphones, the out-of-phase signals would interfere and cancel each other out, leading to an incorrect selection of the quietest tone. Any participant who scored below 83% was rejected from the experiment and did not continue on to the main experimental task. Audio levels in the experiment were set by the participant to a comfortable volume.

The 20 participants who passed the headphone task then completed the main experimental task. To ensure participants could recognize which speaker was the target, an initial familiarization consisted of a set of 20 sentential stimuli with relatively quiet masker speech (+15 dB SNR). Participants were instructed to type each target sentence. The 20 participants then completed the main experimental task, in which the target speech was presented at an SNR of +1 dB.

Performance on the task was calculated by keyword accuracy. Each of the 80 experimental sentences had five keywords, for a total of 400 keywords binarily coded as 1 for Correct and 0 for Incorrect for each participant. Misspelled words were considered incorrect. All versions of any homophones were counted as correct. Finally, participants completed a language background questionnaire containing questions about demographic information and language experience.

The experimental stimuli and procedure in Experiment 1 that manipulated f0 were designed to (1) replicate previous findings in competing speech perception and (2) offer results that can be equitably compared to those in Experiments 2–5 that manipulate speaking rate (Exp. 2), f0 and speaking rate (Exp. 3), and language of the masker (Exps. 4 and 5).

It was predicted that when target and masker share f0 (high f0 talker and high f0 masker, low f0 talker and low f0 masker), accuracy in target speech perception within competing speech will be low. When target and masker mismatch in f0 range (high f0 talker and low f0 masker; low f0 talker and high f0 masker), it was predicted that accuracy in target speech perception within competing speech will be higher.

### 2.4. Results

**Figure 1:**
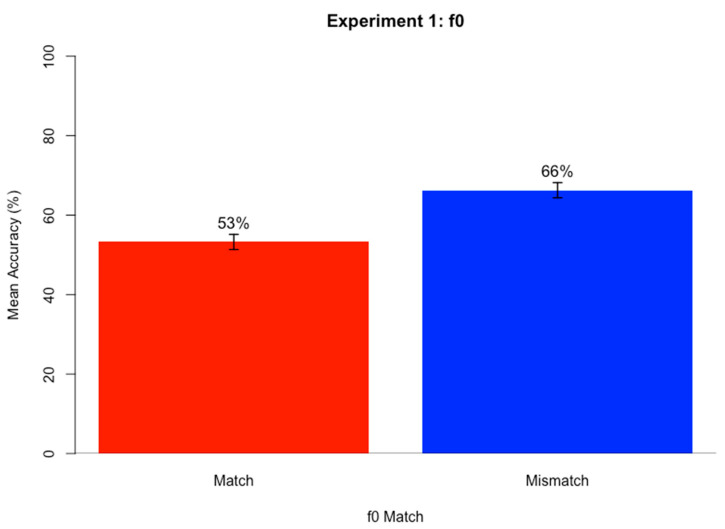
Mean accuracy on sentence transcription task for Experiment 1 when target and masker f0 matched and mismatched. Error bars represent within-participant confidence intervals created using the Cousineau–Morey method [57,58].

Figure 1 shows the mean accuracy on the sentence transcription task when target and masker f0 matched and mismatched. A logistic mixed-effects regression model was run on keyword accuracy scores for the sentence transcription task using the glmer() function of the *lme4* package [55] in R [56] (v. 4.3.0). A best fitting model was identified by stepwise backward likelihood-ratio tests (α = 0.05) using the anova() function in R. The maximal starting model included fixed effects of f0 Match (match vs. mismatch; reference = match) and Target f0 (high vs. low; reference = high) and their interaction, and random intercepts and by-subject and by-item slopes for both fixed effects Target f0 and f0 Match as well as their interaction. F0 Match trials included trials where the target and masker speech both had a high f0 or both had a low f0, and Mismatch trials included trials where the target and masker speech mismatched in f0 (one high and one low f0). Accuracy scores were binarily coded as Correct (coded as 1) and Incorrect (coded as 0). Table 1 contains the best fitting model results.

The significant positive simple effect of f0 Match indicates that accuracy for f0 Mismatch conditions (66%) was significantly higher than for f0 Match conditions (53%). Analysis of each participant’s mean accuracy for f0 Match and f0 Mismatch conditions indicated that all participants had higher accuracy scores for f0 mismatch compared to match conditions.

Target f0 did not remain after backwards fitting, indicating that no statistically significant amount of variance in accuracy scores could be explained by knowing whether target speech had a high or low f0.

#### Discussion

The results of Experiment 1 support the present hypothesis that f0 mismatch conditions yield an advantage over f0 match conditions in competing speech perception. When target and masker shared f0, regardless of whether f0 was high or low, accuracy in target speech perception within competing speech was lower than when target and masker mismatched in f0. This pattern was found for every participant, indicating its robustness.

These results replicate previous findings that a similarity in f0 between target and masker speech makes speech segregation more difficult. This supports what was found when target and masker mismatched in sex [13,14,15], f0 [17], both f0 and vocal tract length [16], and f0 contour [18]. For the present experiment, which varied f0 values for speakers of the same gender, the data clearly show that a similarity in f0 between target and masker speech makes speech segregation much more difficult.

## 3. Experiment 2: The Role of Speaking Rate

Experiment 2 compares fast and slow speaking rates of targets and maskers to identify whether speaking rate behaves in the same way as f0 in competing speech perception in that having a matched speaking rate between target and masker increases masker effectiveness. The present experiment examining speaking rate helps to identify whether there is any difference in masker effectiveness for cues that do not necessarily denote a difference in speaker. f0, which typically denotes a change in speaker when drastically changed uniformly across an entire utterance, differs from speaking rate in that speaking rate can vary considerably within a speaker without denoting a change in speaker. It is possible that these two cues are treated differently during competing speech perception due to their fundamentally different roles in disambiguating talkers. Therefore, it is possible that differences in speaking rate between target and masker will be less effective at aiding in speech segregation compared to f0 differences and may not play a significant role in speech segregation.

### 3.1. Participants

#### Speakers and Listeners

Speakers include the same three female native English speakers of a Midwestern dialect of American English from Experiment 1. The same speaker was used as the target as in Experiment 1.

Listeners included 20 new native English speakers (11 females, 8 males, 1 preferred not to specify; mean age 37.8 years old). Ten were presented with a target talker with a fast speaking rate within slow and fast two-talker masker babble, and the other ten were presented with a target talker with a slow speaking rate within slow and fast two-talker masker babble.

### 3.2. Materials

Stimuli comprised the same subset of the 1965 Revised List of Phonetically Balanced Sentences (Harvard Sentences) [47] as Experiment 1. The procedure to create target speech stimuli within two-talker masker babble was identical to Experiment 1, except with different f0 and speaking rate manipulation values.

Speaking rate was artificially adjusted for all speakers to fall into a fast (6 syllables/second) and a slow (3 syllables/second) range using Praat software [48]. These values were selected to represent values equidistant from the average speaking rate values of the three speakers whose voices were recorded for this experiment (average of 4.5 syllables per second). The speaking rate values selected were played to three phoneticians to ensure they were perceived as natural and comprehensible speaking rate values, and that the slow value was perceived as slow and the fast value was perceived as fast. This resulted in two sets of babble files per masker babble speaker (one for fast speaking rate stimuli and one for slow speaking rate stimuli).

In order to ensure any masking effects are not due to f0 differences, the f0 of all utterances was also adjusted to a neutral value of 200 Hz using the overlap-add feature of Praat [48]. This value was chosen because it falls equidistant between the high f0 and low f0 values chosen for Experiment 1, it is relatively close to the average f0 for the three female speakers’ natural productions (210 Hz), and it was perceived as a natural f0 value for female speech by three phoneticians.

Target files were once again individually manipulated for f0 and speaking rate in the same way as the masker babble files. Each stimulus sentence was created with a fast speaking rate of 6 syllables per second and a slow speaking rate of 3 syllables per second. All stimuli were also manipulated to a neutral f0 value of 200 Hz. Since target speaking rate was a between-subjects variable, all stimulus sentences were created with both a fast and slow speaking rate value so that half of the participants would hear the 100 target stimuli with a fast speaking rate value, and the other half would hear the 100 target stimuli with a slow speaking rate value, thus resulting in 200 target sentence stimuli overall.

All combinations of target and masker speaking rate were created so that there were 50 stimuli with a fast speaking rate target and fast speaking rate masker, 50 stimuli with a fast speaking rate target and slow speaking rate masker, 50 stimuli with a slow speaking rate target and a slow speaking rate masker, and 50 stimuli with a slow speaking rate target and a fast speaking rate masker.

Of the 200 stimuli (160 with an SNR of +1 dB and 40 with an SNR of +15 dB), each participant would hear 100, so that each participant only heard the target as always fast speaking rate or always slow speaking rate within both fast and slow two-talker masker babble. The first 20 stimuli that each participant heard were those with an SNR of +15 dB, and the subsequent 80 stimuli were those with an SNR of +1 dB. The order of presentation of stimuli within the familiarization phase and the experimental phase was randomized across participants.

### 3.3. Procedure

The procedures for Experiment 2 replicated those for Experiment 1.

### 3.4. Results

**Figure 2:**
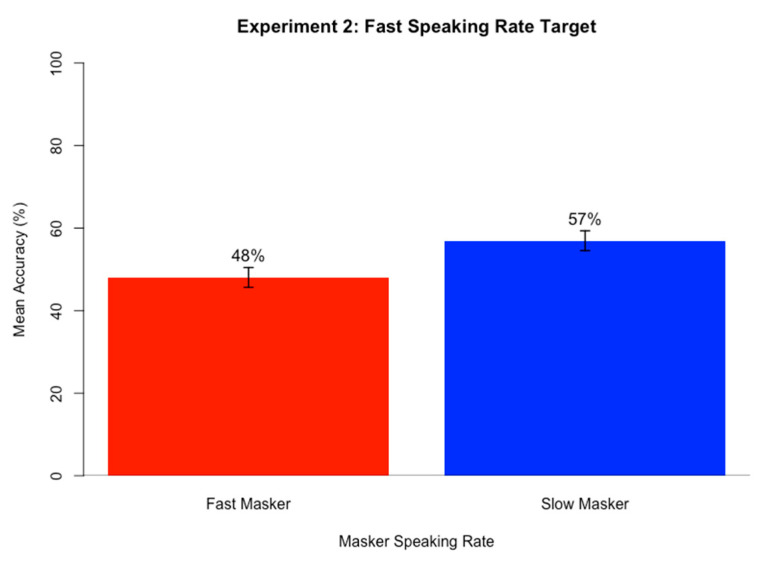

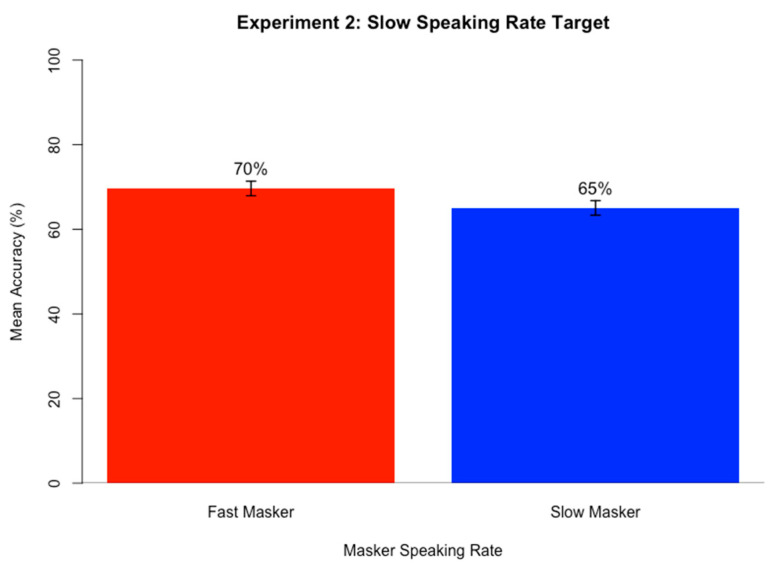
Mean accuracy on sentence transcription task for Experiment 2 when target speaking rate was fast (**top**) and slow (**bottom**) in comparison to masker speaking rate. Error bars represent within-participant confidence intervals created using the Cousineau–Morey method [57,58].

Figure 2 shows mean accuracy on sentence transcription task when target speaking rate was fast (top) and slow (bottom) in comparison to masker speaking rate A logistic mixed-effects regression model was run on keyword accuracy scores for the sentence transcription task using the same approach as in Experiment 1. The fixed effects included Speaking Rate Match (match vs. mismatch; reference = match) and Target Speaking Rate (fast or slow; reference = fast) and their interaction, and random intercepts and by-subject and by-item slopes for both fixed effects Target Speaking Rate and Speaking Rate Match as well as their interaction. Speaking Rate Match trials included trials where the target and masker speech both had a fast speaking rate or both had a slow speaking rate, and mismatch trials included trials where the target and masker speech mismatched in speaking rate (one fast and one slow speaking rate). It was hypothesized that when the target and masker match in speaking rate (either both fast or both slow), keyword accuracy will be the lowest. When the target and masker mismatch in speaking rate, it was predicted that accuracy will be highest. Table 2 contains the best fitting model results after stepwise backward likelihood-ratio testing (α = 0.05). The by-subject random slope for Target Speaking Rate resulted in a singular fit due to a perfect correlation with the intercept, so only random intercepts were retained in the final model, which produced stable and interpretable results without overfitting.

The significant positive simple effect of Speaking Rate Match indicates that accuracy for Speaking Rate Mismatch conditions (63%) was significantly higher than for Speaking Rate Match conditions (57%). Analysis of each participant’s mean Accuracy for Match and Mismatch conditions indicated that 85% of participants had higher accuracy scores for speaking rate mismatch compared to match conditions. The lack of interaction between Speaking Rate Match and Target Speaking Rate indicates this pattern held true when target speech was slow and when it was fast.

The significant positive simple effect of Target Speaking Rate indicates that accuracy was significantly higher for slow target speech conditions (67%) compared to fast speaking rate conditions (53%).

#### Discussion

The results of Experiment 2 support the present hypothesis that mismatch conditions in speaking rate yield an advantage over match conditions in competing speech perception. When target and masker shared speaking rate, regardless of whether it was fast or slow, accuracy in target speech perception within competing speech was lower than when target and masker mismatched in speaking rate. This pattern was found for almost every participant, indicating its robustness. Additionally, the results indicated that fast target trials were harder than slow target trials.

These results mirror those of the f0 data, in that a greater similarity between target and masker in speaking rate leads to more difficult speech segregation. This new finding is in line with the target-masker linguistic similarity hypothesis [38] that claims that the more similar masker speech is to target speech, the more effective masking will be. This holds for a cue, speaking rate, that does not necessarily denote a difference in speaker.

## 4. Experiment 3: f0 and Speaking Rate

It is still unclear how multiple factors interact with each other in competing speech perception. Previous studies have largely been experimentally designed to only evaluate one factor. Because real world speech perception occurs with various maskers which differ from the target in more than one cue, there is a need to systematically investigate how multiple differences between target and masker speech influence masker effectiveness in order to understand how speech perception occurs under ecologically valid degraded conditions.

Van Engen et al. [59] showed that while audiovisual cues, speaking style, and semantic context all improved intelligibility, they also interacted with each other in effectiveness, with, for example, visual information being more helpful for semantically meaningful sentences compared to non-meaningful sentences. While their results demonstrate that different cues interact with each other in their ability to aid with competing speech perception, Van Engen et al. [59] only tested cues that enhance the intelligibility of a speaker (adding semantic context, using clear speech, and adding visual cues), whereas the present study intends to identify the specific relationship between a cue’s role in denoting the presence of a new speaker and its ability to segregate competing speech. The present study thus uses both f0, which typically cues a new speaker, and speaking rate, which typically does not necessarily cue a new speaker, to test this.

In another study that manipulated multiple cues simultaneously, McAuley et al. [22] tested both speech rhythm irregularities and f0 differences in competing speech perception. They found that f0 differences were so salient to listeners that when they were present, speech rhythm irregularities did not have any effect.

Experiment 1 found significant effects of matching versus mismatching f0, and Experiment 2 found significant effects of matching vs. mismatching speaking rate. Because f0 is a cue that typically denotes a change in speaker when hearing an utterance with a low versus a high f0, while speaking rate is a cue that does not necessarily denote a change in speaker when hearing a fast versus a slow utterance, it is possible that these cues will differ in their effectiveness at masking competing speech. The prediction is therefore that there will be a greater ability to segregate target and masker when changing f0 as compared to speaking rate due to f0’s tendency to distinguish between speakers. Additionally, there may be additive effects of differences in both f0 and speaking rate, meaning that the least similarity between target and masker speech will yield the least effective masker, while the most similarity between target and masker speech will yield the most effective masker. However, similar to McAuley et al. [22], it is possible that the high saliency of f0 will mean a cue such as speaking rate does not play a significant role at all in speech perception when both cues are present.

### 4.1. Participants

#### Speakers and Listeners

Speakers included the same three female native English speakers of a Midwestern dialect of American English from Experiment 1. The same speaker was used as the target as in Experiment 1.

Listeners included 90 new native English speakers (29 females, 58 males, 3 preferred not to specify; mean age 36.1 years old). Twenty-three were presented with a target talker with a fast speaking rate and high f0 within slow high, fast high, slow low, and fast low two-talker masker babble, 23 were presented with a target talker with a slow speaking rate and high f0 within slow high, fast high, slow low, and fast low two-talker masker babble, 22 were presented with a target talker with a fast speaking rate and low f0 within slow high, fast high, slow low, and fast low two-talker masker babble, and 22 were presented with a target talker with a slow speaking rate and low f0 within slow high, fast high, slow low, and fast low two-talker masker babble.

### 4.2. Materials

Stimuli were composed of the same subset of the 1965 Revised List of Phonetically Balanced Sentences as in Experiments 1 and 2. All procedures for creating experimental stimuli are the same as Experiments 1 and 2 except for the f0 and speaking rate manipulations.

Speaking rate was adjusted for all speakers to fall into a fast (6 syllables/second) and a slow (3 syllables/second) range using Praat software [48]. The f0 of all utterances was also artificially adjusted for all speakers to fall into a high f0 (225 Hz) and a low f0 (175 Hz) range using the speech manipulation software, Praat [48]. In the experiment, target speaking rate and f0 were a between-subjects variable. All stimulus sentences were created with a high f0 and fast speaking rate, a low f0 and fast speaking rate, a high f0 and slow speaking rate, and a low f0 and slow speaking rate value since one fourth of the participants would hear the 100 stimuli with a high f0 and fast speaking rate value, one fourth would hear a low f0 and fast speaking rate, one fourth would hear a high f0 and slow speaking rate, and one fourth would hear the 100 stimuli with a low f0 and slow speaking rate value, thus resulting in 400 target sentence stimuli overall. Masker speech was also composed of all combinations of f0 and speaking rate, thus resulting in 4 masker speech files to create the competing speech stimuli.

Stimuli were created for all combinations of target and masker f0 and speaking rate so that there were 16 types of stimuli (4 target types * 4 masker types). Each participant heard only one of the target types throughout the experiment within all of the four masker types.

### 4.3. Procedure

The procedures for the experiment replicated those for Experiment 1. For 80 of the participants, the experiment was conducted remotely online using Gorilla Experiment Builder [53]. In order to assess whether performance would be similar for in-person and online participants, an additional ten participants completed the experiment at the KU Phonetics and Psycholinguistics Lab using identical procedures as those who completed the experiment remotely. All participants who participated in Experiment 3 also participated in either Experiment 4 (replication of Experiment 3 but with Dutch masker babble) or Experiment 5 (replication of Experiment 3 but with French masker babble). See Section 5 and Section 6 for details on these experiments.

### 4.4. Results

A logistic mixed-effects regression model was run on keyword accuracy scores using the same approach as in Experiments 1 and 2. Fixed effects included f0 Match (match vs. mismatch; reference = match) and Speaking Rate Match (match vs. mismatch; reference = match) and their interaction, and random intercepts and by-subject and by-item slopes for both fixed effects Speaking Rate Match and f0 Match as well as their interaction. f0 Match trials included trials where the target and masker speech both had a high f0 or both had a low f0, and Mismatch trials included trials where the target and masker speech mismatched in f0 (one high and one low f0). Speaking Rate Match trials included trials where the target and masker speech both had a fast speaking rate or both had a slow speaking rate, and Mismatch trials included trials where the target and masker speech mismatched in speaking rate (one fast and one slow speaking rate). It was hypothesized that when the target and masker match in both f0 and speaking rate, keyword accuracy will be the lowest, whereas when target and masker mismatch in both f0 and speaking rate, keyword accuracy will be the highest. Table 3 contains the best fitting model results after stepwise backward likelihood-ratio testing (α = 0.05).

**Figure 3:**
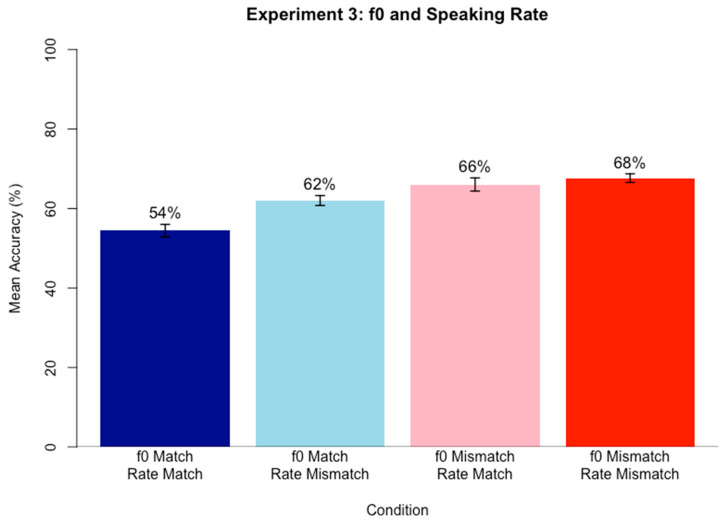
Mean accuracy on sentence transcription task for Experiment 3 for all participants by f0 and speaking rate conditions; Error bars represent within-participant confidence intervals created using the Cousineau–Morey method [57,58].

The significant positive simple effect of f0 Match indicates that accuracy for f0 Mismatch conditions was higher than for f0 Match conditions. Figure 3 shows the pattern, with mean accuracy values in f0 mismatch conditions higher than in f0 match conditions. The final model retained the simple effect of Speaking Rate Match for theoretical completeness, although this effect was not statistically significant, meaning it was not a significant predictor of accuracy scores. An alternative version of the model was also created without backwards fitting, and again, the fixed effect of Speaking Rate Match did not reach significance. Neither of the models revealed a simple effect of Speaking Rate Match.

#### Discussion

The results of Experiment 3 support the hypothesis that mismatch conditions yield an advantage over match conditions in competing speech perception. However, the relationship seems to only depend on f0 Match, not Speaking Rate Match. When target and masker share f0, regardless of whether it is high or low, accuracy in target speech perception within competing speech was lower than when target and masker mismatched in f0. In contrast, Speaking Rate match did not explain a significant amount of variance in accuracy scores, meaning that whether speaking rate matched or mismatched between target and maskers could not predict performance on the task.

The results may initially seem surprising given the results of Experiment 2, which found a significant effect of Speaking Rate Match. However, McAuley et al. [22] showed a similar set of results in that while rhythm irregularities could influence target speech perception during competing speech perception, when a highly salient cue like f0 was present, speech rhythm irregularities no longer had an effect. While the way they created speech rhythm irregularities is not the same as the speaking rate manipulations of the present study, the results suggest a similar explanation, whereby f0 is salient enough that speaking rate effects no longer matter. These results would also make sense given that f0 is a cue that seems to be encoded in memory as a reliable means of identifying talker identity, while speaking rate may not be encoded in memory since it is less reliable. The highly salient and reliable f0 cue seems to wash away any effect of speaking rate that may occur. Similarly, when the f0 cue was not available (in f0 Match conditions), there was a significant difference in accuracy scores depending on speaking rate (with higher accuracy for a mismatch in speaking rate compared to a match in speaking rate). This finding again points to the same conclusion that speaking rate differences can benefit competing speech perception, but not when a salient cue like f0 is already available to aid in segregation.

The data do seem to trend in the predicted direction of gradual increases in accuracy as similarity between target and masker speech decreased. For example, when both f0 and Speaking Rate matched, accuracy was lowest (54%), but when only f0 or only speaking rate matched, accuracy was higher (62% and 66%, respectively). Finally, when both speaking rate and f0 mismatched, accuracy was highest (68%). These results again align with the target-masker linguistic similarity hypothesis [38].

Overall, the data indicate a differential role of f0 compared to speaking rate, with f0 performing as the more salient cue that, when present and mismatching, makes speaking rate inconsequential. This suggests a different role between the two acoustic cues during speech segregation, likely that the cue more able to distinguish between speakers is the one listeners attend to.

## 5. Experiment 4: f0 and Speaking Rate with Dutch Masker Speech

The similarity between and knowledge of target and masker languages can also influence performance. When an unknown language masker is more similar to the target, such as Dutch masking English, it is a more effective masker than when a dissimilar language masks a target, such as Mandarin masking English [39]. The rhythm class of a language can also influence competing speech perception. A mismatch in rhythm class between the target and masker can lead to less effective masking [24]. These findings all lend support to the idea that increasing similarity between the target and masker speech leads to increased difficulty in segregating speech streams [38]. 

Robust evidence from previous research also indicates that not knowing the language of the masker speech significantly improves target speech perception and reduces masker effectiveness [24,35,36,37,38,39,40,41,42].

In order to test whether the use of f0 and speaking rate cues differs when an unknown language is used for the masker speech and to create results that can be directly compared to planned follow up tests on the similarity between target and masker languages, this experiment replicates Experiment 3 with English target speech and Dutch masker speech. Dutch was selected due to its similarity to English in rhythm class and sound inventory. Both English and Dutch are stress-timed languages [27].

Experiment 4 will identify how language knowledge, f0, and speaking rate interact in influencing target speech perception in competing masker speech. Results of this study inform whether f0 and speaking rate interact differently during competing speech perception in unknown (yet rhythmically matched) masker speech. It is possible that f0 and speaking rate do not matter when the masker speech is an unknown language. Compared to English masker babble (Experiment 3), Dutch masker babble may be a much less effective masker, and other acoustic properties of the target and masker speech may not play a role in speech segregation. If this is the case, accuracy should be similar regardless of the f0 and speaking rate differences between target and masker speech. English and Dutch both share many rhythm properties, so it is still possible that Dutch masker speech may cause some degree of masking that is modulated by the acoustic properties of the masker speech. It was hypothesized again that the more similar the target and masker are in terms of f0 and speaking rate, the lower the accuracy. In particular, if target and masker are both high f0 and fast speech, then accuracy will be lower compared to if the target and masker share f0 but not speaking rate or if they do not share either f0 or speaking rate. It is also possible that f0 may more drastically impact perception compared to speaking rate since f0 is more likely to cue a different speaker compared to speaking rate. As a result, it is possible that when f0 differs between target and masker, accuracy will be high, and when f0 is similar for target and masker speech, accuracy will be low, regardless of speaking rate.

### 5.1. Participants

#### 5.1.1. Speakers

Speakers included the same female native English speaker of a Midwestern dialect of American English who was the target speaker from Experiments 1–3. The masker speakers for this experiment were two female native Dutch speakers (ages 23 and 28) living in the Netherlands at the time of the recordings (both from Amsterdam).

#### 5.1.2. Listeners

Participants included a subset of 45 of the native English speakers (12 females, 31 males, 2 preferred not to specify; mean age 36.0 years old) from Experiment 3 who were not familiar with Dutch.

### 5.2. Materials

Two-talker masker babble was presented in Dutch, and target speech was presented in English. Target stimuli were composed of the same subset of the 1965 Revised List of Phonetically Balanced Sentences (Harvard Sentences) [47] as Experiment 3. All procedures for stimulus creation are identical to Experiment 3, except the masker babble is now in Dutch instead of English. The Dutch babble sentences were translations of the IEEE sentences (kindly provided by Dr. Susanne Brouwer).

Speaking rate was artificially adjusted for all speakers to fall into a fast (6 syllables/second) and a slow (3 syllables/second) range using Praat software [48]. The f0 of all utterances was also artificially adjusted for all speakers to fall into a high f0 (225 Hz) and a low f0 (175 Hz) range using Praat [48].

### 5.3. Procedure

All participants who completed Experiment 4 completed Experiment 3 first. The experiment was conducted remotely online using Gorilla Experiment Builder [53]. Since the target speaker remained the same as in Experiment 3, no new familiarization phase was needed. Participants completed 80 experimental trials.

### 5.4. Results

A logistic mixed-effects regression model was run on keyword accuracy scores for the sentence transcription task using the same approach as in Experiment 3. Table 4 contains the best fitting model results after backward likelihood-ratio testing (α = 0.05).

**Figure 4:**
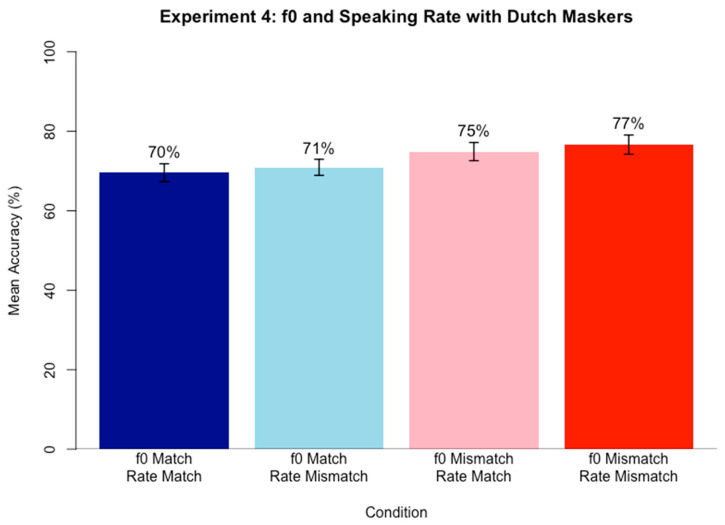
Mean accuracy on sentence transcription task for Experiment 4 for all participants when listening to English target speech and Dutch masker speech by f0 and speaking rate conditions. Error bars represent within-participant confidence intervals created using the Cousineau–Morey method [57,58].

The significant positive simple effect of f0 Match indicates that accuracy for f0 Mismatch conditions was higher than for f0 Match conditions within Dutch masker speech. Figure 4 demonstrates the pattern, with mean accuracy values in f0 Mismatch conditions (pink and red) higher than in match conditions (blue and light blue). Speaking Rate Match did not remain after backwards fitting, indicating that no statistically significant amount of variance in accuracy scores could be explained by the Speaking Rate Match status of the stimuli within Dutch masker speech. An alternative version of the model was also created without backwards fitting; again, the fixed effect of Speaking Rate Match did not reach significance. Neither of the models revealed a simple effect of Speaking Rate match.

#### Discussion

The results of Experiment 4 (containing Dutch maskers) mirror those of Experiment 3 (containing English maskers). They support the present hypothesis that mismatch conditions yield an advantage over match conditions in competing speech perception with an unknown language masker, although the relationship seems to only depend on f0, not speaking rate. When target and masker share f0, regardless of whether it is high or low, target speech perception within competing speech is less accurate than when target and masker mismatch in f0. Speaking rate did not explain a significant amount of variance in accuracy scores, meaning that whether speaking rate matched or mismatched between targets and maskers could not predict performance on the task. Even when no f0 cue was available to aid in speech segregation, the difference in accuracy between Speaking Rate Match and Speaking Rate Mismatch was not significant.

The data again pattern in the predicted direction of gradual increases in accuracy as similarity between target and masker speech decreases, although the pattern is not significant. Specifically, when both f0 and Speaking Rate matched, accuracy was lowest (70%), but when only f0 or only Speaking Rate matched, accuracy was higher (71% and 75%, respectively). Finally, when both Speaking Rate and f0 mismatched, accuracy was highest (77%). These results again align with the target-masker linguistic similarity hypothesis [38], although results must be interpreted with caution with respect to speaking rate since the pattern is not significant.

Overall, the data indicate a differential role of f0 compared to speaking rate, with f0 performing as the more salient cue that, when present, renders speaking rate inconsequential. This pattern remains the same even when the masker speech is an unknown, but rhythmically similar, language, Dutch.

## 6. Experiment 5: f0 and Speaking Rate with French Masker Speech

Another factor that may influence competing speech perception is rhythm class. Reel and Hicks [24] suggest that the rhythm class of a language may influence its effectiveness at masking target speech. Similarly, Calandruccio and Zhou [23] found that English/Greek bilinguals still showed greater masker effectiveness when the target and masker were the same language even when listeners understood both languages, either because of rhythm class differences between English and Greek or because of differences in speaking rate between both languages. Since that study’s methodology precluded the ability to disambiguate between those two possible explanations, the present study intended to explicitly test whether masker effectiveness is driven more by speaking rate or by rhythm class properties.

**Figure 5:**
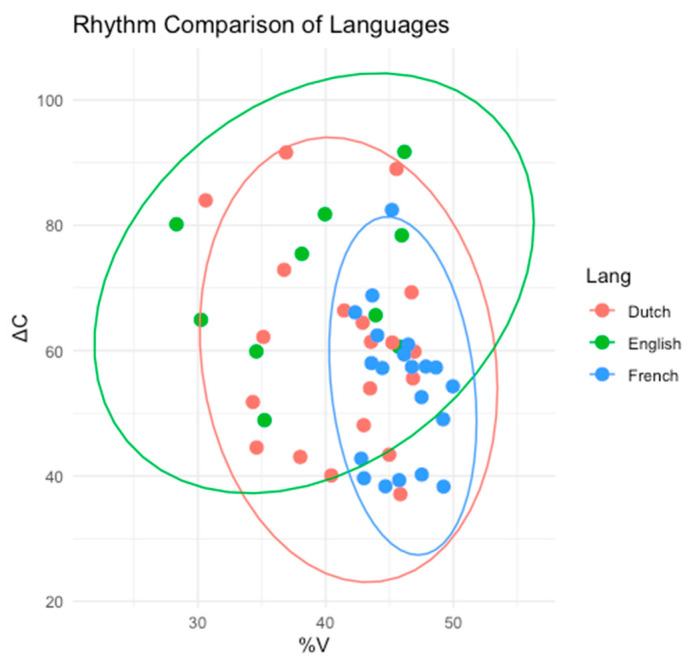
Scatterplot of %V and ΔC for a subset of 50 sentences across Dutch, English, and French; Ellipses represent 95% confidence regions around the group centroids, based on the pooled covariance matrix within each language.

In order to test this, masker effectiveness is compared for masker languages with the same stress-timed rhythm class as English (Dutch in Experiment 4) and masker languages with a different rhythm class from English (French in Experiment 5). French was selected as the masker language with a different rhythm class because it is a syllable-timed language [27]. In order to experimentally determine whether the present study’s stimuli demonstrate the expected stress-timed and syllable-timed differences among the three languages, %V and ΔC were plotted for a subset of 10 randomly selected English sentences produced by the target speaker, 10 randomly selected sentences produced by each Dutch masker, and 10 randomly selected sentences produced by each French masker, for a total of 50 sentences. Figure 5 shows the differences across languages.

A one-way multivariate analysis of variance (MANOVA) was conducted to examine whether the combination of %V and ΔC differed depending on the language (English, Dutch, or French). Using Pillai’s trace, there was a significant multivariate effect of Language, *V* = 0.388, *F* (4, 94) = 5.65, *p* < 0.001.

Follow-up pairwise MANOVAs revealed that English and Dutch did not differ significantly, (*p* = 0.132); however, French differed significantly from both English (*p* < 0.001) and Dutch (*p* = 0.002). These results suggest that French exhibits a distinct rhythm pattern compared to the other two languages, which follows expectations from previous literature.

The present study attempted to determine whether speaking rate influences masker effectiveness more for Dutch compared to French masker speech, or whether the role of speaking rate does not depend on rhythm class. If masker effectiveness is driven more by speaking rate, it would be expected that both Dutch and French maskers show similar patterns of speaking rate’s effectiveness at segregating the speech signal, regardless of rhythm class. If masker effectiveness is driven more by rhythm class properties, it would be expected that Dutch and French maskers will differ in terms of how they are influenced by speaking rate; it is likely that Dutch causes more interference with similar speaking rates for target and masker speech compared to French. Alternatively, it is also possible that neither speaking rate nor rhythm class effects may appear in the results. Based on the target–masker similarity hypothesis that states that more similarity between target and masker will lead to a greater masker effectiveness [38], it is hypothesized that there will be a greater influence of speaking rate for the same rhythm class language masker compared to the different rhythm class masker.

Additional differences between Dutch and French beyond just rhythm properties may involve their sound inventories and the similarity of these inventories to English. While all three languages share a number of overlapping sounds, there are also differences across the languages [32,60,61]. While English and Dutch both allow for diphthongs, the exact diphthongs that appear differ across languages. Meanwhile, French does not allow for diphthongs. While English does not contain rounded front vowels, Dutch contains two, and French contains three. Both Dutch and French also contain uvular consonants that English does not have. Additionally, the same phoneme may also be produced in an acoustically different way depending on the language. For example, while all three languages distinguish between voiced and voiceless stops, voiced stops in English are sometimes realized as voiceless unaspirated stops in word-initial position [62], but voiced stops in French and Dutch are typically realized as fully voiced [32,60]. Overall, English, Dutch, and French vary in similarity to each other in terms of multiple cues beyond rhythm properties, such as phoneme inventory (diphthongs, uvular consonants, rounded front vowels) and the phonetic realization of sounds (voiced versus voiceless unaspirated stops).

### 6.1. Participants

#### 6.1.1. Speakers

Speakers included the same female native English speaker of a Midwestern dialect of American English who was the target speaker from Experiments 1–4. The masker speakers for this experiment were two female native French speakers (ages 32 and 29 years old) from France (one from Croix and the other from Grenoble).

#### 6.1.2. Listeners

Participants included a subset of 45 of the native English speakers (17 females, 27 males, 1 preferred not to specify; mean age 36.2 years old) from Experiment 3 who were not familiar with French. Two-talker masker babble was presented in French, and target speech was presented in English.

### 6.2. Materials

Stimuli were composed of the same subset of the 1965 Revised List of Phonetically Balanced Sentences (Harvard Sentences) [47] as Experiment 3. All procedures for stimulus creation are identical to Experiment 3 except the masker babble is now in French instead of English. A set of 165 phonemically balanced French sentences inspired by the Harvard Sentences were used for the French babble [63].

Speaking rate was adjusted for all speakers to fall into a fast (6 syllables/second) and a slow (3 syllables/second) range using Praat [48]. The f0 of all utterances was also adjusted for all speakers to fall into a high f0 (225 Hz) and a low f0 (175 Hz) range using Praat [48].

### 6.3. Procedure

The procedures for the experiment replicated those for Experiment 3. For 35 of the participants, the experiment was conducted remotely online using Gorilla Experiment Builder [53]. Ten additional participants completed the experiment at the KU Phonetics and Psycholinguistics Lab using identical procedures as those who completed the experiment remotely.

After completing Experiment 3, participants completed Experiment 5. Since the target speaker remained the same as in Experiment 3, no new familiarization phase was needed. Participants completed 80 experimental trials.

### 6.4. Results

A logistic mixed-effects regression model was run on keyword accuracy scores for the sentence transcription task using the same approach as in Experiment 4. It was hypothesized that when the target and masker match in both f0 and speaking rate, keyword accuracy will be the lowest, whereas when target and masker mismatch in both f0 and speaking rate, keyword accuracy will be the highest.

Table 5 contains the best fitting model results after backward likelihood-ratio testing (α = 0.05).

**Figure 6:**
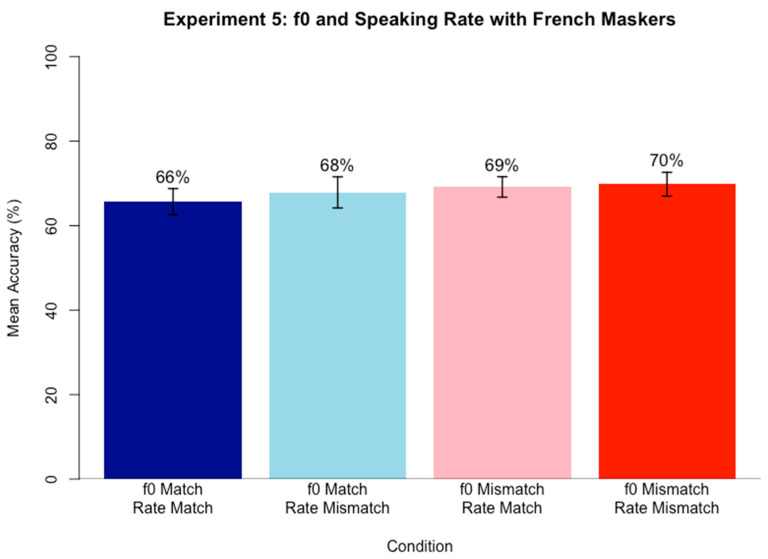
Mean accuracy on sentence transcription task for Experiment 5 for all participants when listening to English target speech and French masker speech by f0 and Speaking Rate Match conditions; Error bars represent within-participant confidence intervals created using the Cousineau–Morey method [57,58].

The final model retained fixed effects of f0 Match and Speaking Rate Match, and random slopes for both predictors by Subject and Item. While neither fixed effect was statistically significant in the final model (*p* > 0.05), model comparisons during backward fitting confirmed that both were necessary to retain, suggesting considerable inter-individual and item-level variability in their influence on accuracy. These results indicate that no statistically significant amount of variance in accuracy scores could be explained by the f0 Match or Speaking Rate Match status of the stimuli in French masker speech. Figure 6 shows the results.

To determine whether the lack of an effect was due to failure to compare to the proper baseline, the logistic mixed-effects regression model was releveled to all possible baseline combinations for f0 Match and Speaking Rate match and rerun. None of the models revealed any significant effects.

#### 6.4.1. Comparison of Experiments 3 (English), 4 (Dutch), and 5 (French)

To identify whether performance differed by masker language, a logistic mixed-effects regression model was run on keyword accuracy scores for the sentence transcription task in Experiments 3, 4, and 5. Fixed effects included f0 Match (match vs. mismatch; reference = match), Speaking Rate Match (match vs. mismatch; reference = match), and Language (English, Dutch, French; reference = Dutch) and their interactions, random intercepts of Subject and Item were included, and by-subject slopes for all fixed effects (Speaking Rate Match, f0 Match, and Language) were included. Random slopes for Language by Subject were not included because Language was between-subjects in the combined dataset and therefore did not vary within individuals (participants who completed both Experiment 3 and Experiment 4 or Experiment 3 and Experiment 5 were treated as distinct subjects for each sub experiment in this combined model in order to avoid conflating individual people’s variability with their variability resulting from experimental differences). By-item random slopes and interactions in the by-subject random slopes were not included to achieve model convergence and interpretability. It was hypothesized that accuracy would be higher when the masker was an unknown language compared to English due to the lack of informational masking present when the masker language is unknown. Table 6 contains the best fitting model results after backward likelihood-ratio testing (α = 0.05).

**Figure 7:**
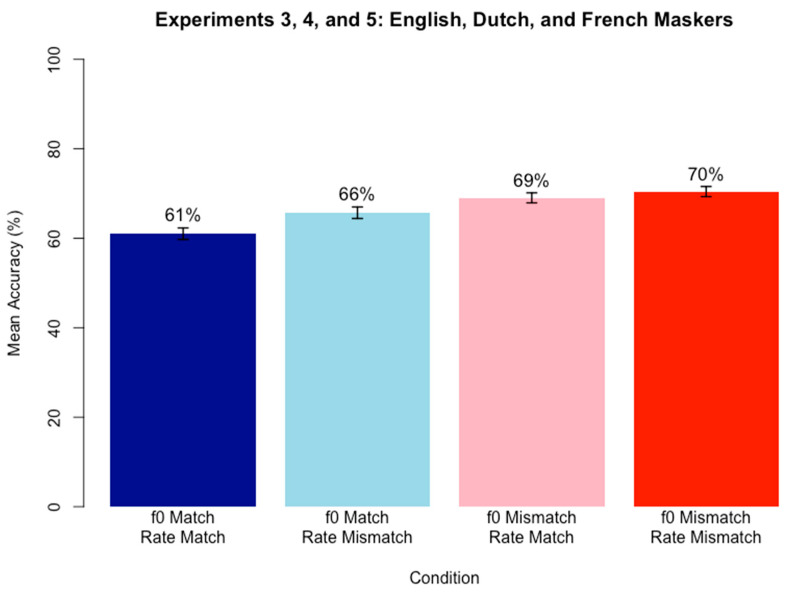
Mean accuracy on sentence transcription task for Experiments 3, 4, and 5 for all participants when listening to English target speech and pooled across masker language by f0 and Speaking Rate Match conditions. Error bars represent within-participant confidence intervals created using the Cousineau–Morey method [57,58].

The significant positive simple effect of f0 Match indicates that accuracy for f0 Mismatch conditions was significantly higher than for f0 Match conditions when the masker language was Dutch. The lack of interaction between f0 Match and Language suggests this pattern remains the same for stimuli with English and French maskers. This means that regardless of the masker language (English, Dutch, or French), participants were more accurate when target and masker speech mismatched in f0 than when they matched. While Speaking Rate Match was retained during backward fitting, it did not reach significance in the final model, indicating that no statistically significant amount of variance in accuracy scores could be explained by the Speaking Rate Match status of the stimuli. Figure 7 shows the results.

**Figure 8:**
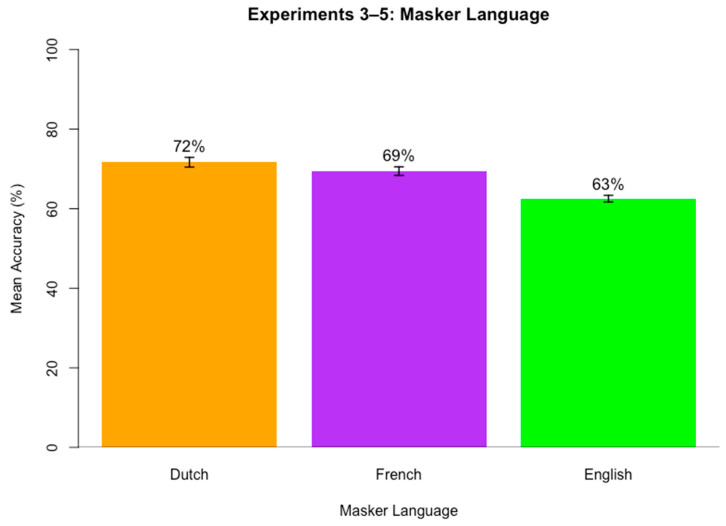
Mean accuracy on sentence transcription task for Experiments 3, 4, and 5 for all participants by Masker Language; Error bars represent within-participant confidence intervals created using the Cousineau–Morey method [57,58].

The significant negative simple effect of Language indicates that accuracy for English was lower than for Dutch for f0 Match conditions. The lack of interaction between f0 Match and Language suggests this patten remains the same for f0 Mismatch conditions. The significant negative simple effect of Language indicates that accuracy for French conditions was lower than for Dutch conditions for f0 Match conditions. The lack of interaction between f0 Match and Language suggests this patten remains the same for f0 Mismatch conditions. The model was releveled (baseline = English) to compare English and French, and a significant positive simple effect of Language was found (*β* = 0.337, SE = 0.107, *t* = 3.147, *p* = 0.002), indicating that accuracy for French conditions was higher than for English conditions for f0 Match conditions. The lack of interaction between f0 Match and Language suggests this patten remains the same for f0 Mismatch conditions. These results indicate that accuracy was highest when identifying target speech within Dutch masker speech, lower when identifying target speech within French masker speech, and lowest when identifying target speech within English masker speech, regardless of whether f0 matched or mismatched for the targets and maskers. Figure 8 illustrates the pattern.

To identify whether performance differed for participants tested online and in-person, the best fitting model for Experiments 3, 4, and 5 (Accuracy ~ f0 Match + Language + Speaking Rate Match + (1 + f0 Match + Speaking Rate Match|Subject) + (1|Item)) was compared to a model also containing Online status as a fixed effect (Accuracy ~ f0 Match + Language + Online + Speaking Rate Match + (1 + f0 Match + Speaking Rate Match|Subject) + (1|Item)). A single term deletion analysis was conducted on the more complex version of the model. The analysis revealed that eliminating the Online term did not significantly impact model fit (χ^2^ (1) = 0.072, *p* = 0.789), indicating that whether participants completed the experiment online or in-person did not seem to influence their accuracy. This means that participants performed similarly on the task regardless of whether they completed the experiment in-person or remotely online.

#### 6.4.2. Discussion

The results of Experiment 5 (with French maskers) failed to reach significance for simple effects of f0 Match and Speaking Rate Match. The data do not show any difference in performance for different combinations of f0 and speaking rate for targets and maskers. Participants generally performed similarly regardless of the f0 and speaking rate characteristics of the stimuli.

The data again trend in the predicted direction of gradual increases in accuracy as the similarity between target and masker speech decreased. For example, when both f0 and speaking rate matched, accuracy was lowest (66%), but when only f0 or only speaking rate matched, accuracy was higher (68% and 69%, respectively). Finally, when both speaking rate and f0 mismatched, accuracy was highest (70%). These results again align with the target-masker linguistic similarity hypothesis [38].

When conducting analyses on Experiments 3, 4, and 5 (English versus Dutch versus French), the data support the hypothesis that mismatch conditions yield an advantage over match conditions in competing speech perception, although the relationship seems to only depend on f0, not speaking rate. When target and masker share f0, regardless of whether it is high or low, accuracy in target speech perception within competing speech is lower than when target and masker mismatch in f0. In contrast, speaking rate did not explain a significant amount of variance in accuracy scores, meaning whether speaking rate matched or mismatched between targets and maskers could not predict performance on the task.

Overall, the data indicate a differential role of f0 compared to speaking rate, with f0 performing as the more salient cue that, when present, makes speaking rate unimportant. This pattern was observed when masker speech was in English and Dutch, but not when masker speech was in French. While the Experiment 5 data numerically patterned in the predicted direction, with performance highest for fully mismatching stimuli and performance lowest for fully matching stimuli, the simple effects of f0 Match and Speaking Rate Match did not reach significance when French maskers were used.

## 7. Discussion and Conclusions

The aim of this study was to examine the role of f0, speaking rate, and rhythm in competing speech perception. To achieve this, five sentence transcription tasks were conducted, in which target speech was presented within two-talker masker babble. In the first experiment, the voices of three female speakers were adjusted to have either high or low f0 values to determine whether an f0 mismatch between target and masker speech would facilitate speech perception. The second experiment similarly manipulated the speakers’ voices to have either a fast or slow speaking rate to assess whether a difference in speaking rate would reduce masking effects. The third experiment combined manipulations of both f0 and speaking rate to explore their interaction. In the fourth experiment, Dutch—a language with rhythmic properties similar to the target speech—was used as the masker to examine whether reduced lexical competition would aid speech segregation. Lastly, the fifth experiment introduced French, a language with distinct rhythmic properties from the target speech, to investigate the influence of rhythm on speech segregation.

Experiment 1 found clear evidence of release from masking when participants heard a target and masker mismatching in f0 compared to when they heard a target and masker with the same f0. Results from Experiment 2 indicated that when target and masker speech mismatched in speaking rate, accuracy was better than when they matched. This indicates f0 and speaking rate seem to behave in the same way during competing speech perception, and the results give further evidence for the target-masker linguistic similarity hypothesis [38], which claims that as the similarity between target and masker increases, masking is more effective. This holds true for both f0 and speaking rate when examined separately.

One reason why the fast target was more difficult than the slow target is likely due to the less taxing cognitive processing required to disambiguate a slow speech signal, which gives more time as it unfolds to identify the target and process its contents.

In order to determine whether f0 and speaking rate interact, both f0 and speaking rate were manipulated for the target and masker speech in Experiment 3. The results for Experiment 3 indicated that only f0 seemed to matter when both f0 and speaking rate varied, as indicated by the simple effect of f0 Match without any simple effect or interactions for Speaking Rate Match. These results are in line with previous research by McAuley et al. [22], which found that when a salient cue such as f0 was present, rhythm irregularities no longer influenced accuracy.

When comparing accuracy scores between Speaking Rate Match and Mismatch conditions for the subset of data with matching f0 values between target and masker speech, there was significantly higher accuracy for Speaking Rate Mismatch conditions. In contrast, there was no significant difference in accuracy scores depending on speaking rate properties. This indicates that speaking rate differences between target and masker speech only seem to matter when f0 cues do not contribute and conflict.

Results from Experiments 1, 2 and 3 indicate that, while both f0 and speaking rate differences between target and masker speech offered release from masking when only one cue (either f0 or speaking rate) was manipulated, when both cues were manipulated, f0 and speaking rate behaved differently. f0, the cue that more effectively indicates a change in talker, became the only relevant cue to influence release from masking. In comparison, speaking rate, the cue that is not as effective in signaling a change in talker, did not seem to matter when f0 cues were present. These results suggest that the ability for a cue to identify a speaker may influence how influential it is when trying to segregate multiple speech streams.

While evidence for additive effects of both f0 and speaking rate in release from masking did not reach significance, the data numerically patterned in the predicted direction, with accuracy highest for target and masker speech combinations that were most different (mismatched in both f0 and speaking rate), accuracy in the middle for target and masker speech that differed in only one cue (mismatched in either f0 or speaking rate), and accuracy lowest for target and masker speech that differed in no cues (matched in both f0 and speaking rate). This general pattern does align with the predictions of the target-masker linguistic similarity hypothesis [38] in that as the similarity between target and masker increases, accuracy decreases. This offers some degree of evidence that there can be additive effects of both f0 and speaking rate over just f0 or just speaking rate alone in aiding with speech segregation, although the lack of significance of these findings means no firm conclusions can be drawn.

To identify whether participants still pay attention to f0 and speaking rate in the same way when masker speech is in an unknown language, and thus does not cause additional lexical competition, Experiment 4 replicated Experiment 3 using Dutch masker babble instead of English masker babble. Dutch and English are both stress-timed languages, meaning both languages share similar rhythm properties. Results indicated that participants behaved similarly in their use of f0 and speaking rate cues as in Experiment 3 with English masker babble.

To determine whether the unknown language masker was less effective than the known language masker, a comparison of the results of Experiments 3 and 4 demonstrated that accuracy was higher for Dutch compared to English for all conditions, as indicated by a simple effect of Language (*β* = −0.624, SE = 0.105, *t* = −5.968, *p* < 0.001) with no interactions between Language and either f0 Match or Speaking Rate Match. These findings may result from the lack of lexical competition and informational masking that the unknown language masker has compared to English. Additionally, it could be that there are other acoustic differences between Dutch and English that may make Dutch a less effective masker, such as differences in phoneme inventory.

Previous research focusing on quantifying the similarity between languages has used various measures to do so. For example, Eden [64] measured distance between languages based on how many linguistic parameters differ between languages and used probabilistic models of one language to predict the upcoming sound or letter in another language. Eden [64] used a multitude of parameters when comparing English, Dutch, and French, including phonotactic constraints and syllable structure properties, as well as vowel and consonant inventories. Any of these different parameters can contribute to the degree of masker effectiveness during competing speech perception. Based on these parameters, English and Dutch were classified as closer than English and French or Dutch and French. While these parameters did not involve many rhythm-related features of the languages, these measures still offer some means by which to quantify linguistic similarity between languages.

Experiment 5 replicated Experiment 3, except with French masker speech instead of English masker speech. The goal of this experiment was to identify whether a different unknown language masker with rhythm properties that differ more between target and masker speech would still cause participants to use f0 and speaking rate in the same way, with particular focus on how speaking rate’s influence may differ for languages of different rhythm classes. The results, however, demonstrated no significant effects, meaning neither the f0 nor the speaking rate of the stimuli predicted performance on the task. While no significant effects were found, results again numerically offered some evidence in favor of the target-masker linguistic similarity hypothesis [38], with accuracy highest for the most distinct target-masker pairs (f0 and speaking rate both mismatching) and accuracy lowest for the most similar target-masker pairs (f0 and speaking rate both matching).

The lack of significant findings for Experiment 5 may possibly result from French being a poor masker that participants are good at ignoring regardless of other acoustic cues available to aid in speech segregation. This would potentially mean participants can achieve good accuracy without needing to pay attention to acoustic cues such as f0 or speaking rate.

A model combining Experiments 3 (English masker speech) and 5 (Dutch masker speech) established that the unknown language masker advantage found for Dutch also occurred for French. Specifically, the significant simple effect of Language without any interactions with f0 Match or Speaking Rate Match (*β* = 0.339, SE = 0.108, *t* = 3.133, *p* = 0.002) indicated that participants were more accurate when the masker was an unknown language, French, compared to a known language. This again aligns with previous literature that unknown language maskers are typically less effective than known language maskers [8,23,24,35,36,37,38,39,40,41,42].

Experiments 4 (Dutch masker speech) and 5 (French masker speech) were also compared. This analysis found no effect of Language (Language did not remain after backwards fitting the model), indicating that while participants experienced greater accuracy for unknown masker speech (either Dutch or French) compared to known masker speech (English), the actual masker language itself did not matter.

Further statistical analyses were conducted for Experiments 3, 4, and 5 to identify the role of masker background language in how rhythm and linguistic knowledge influence speech segregation. When data from Experiments 3 and 5 or from Experiments 4 and 5 were combined, the simple effect of f0 Match consistently remained (*β* = 0.411, SE = 0.107, *t* = 3.830, *p* < 0.001, and *β* = 0.400, SE = 0.153, *t* = 2.613, *p* = 0.009, respectively), and no effect or interaction of Speaking Rate Match was found (Speaking Rate Match did not remain after backwards fitting). Because nearly all models (those containing the results from Experiments 1, 3, 4, 3+4, 3+5, and 4+5) except for Experiment 5 alone contained the simple effect of f0 Match without any effect of speaking rate, the salient role of f0 in competing speech perception and the lack of role of speaking rate when f0 is present appear to be relatively robust patterns.

These results become more complicated when running an analysis on Experiments 3, 4, and 5 together. In this analysis, there was an effect of language that indicated all three masker languages significantly differed in accuracy scores, with English maskers resulting in the poorest accuracy (most effective masking), French maskers resulting in the next poorest accuracy, and Dutch maskers resulting in the best accuracy (least effective masking). While English maskers were expected to be most effective, the advantage for Dutch maskers over French maskers was more surprising. Since Dutch and English share a rhythm class while French does not, it was expected that Dutch would be a more effective masker than French, but the opposite result was found. However, since these results did not hold when just comparing Dutch and French alone (Experiments 4 + 5), the finding may not be robust.

Another goal of the current study was to identify whether the role of speaking rate differed for Dutch masker stimuli compared to French masker stimuli due to their differences in rhythm properties. It was predicted that speaking rate differences between target and masker speech would be more impactful for Dutch masker speech compared to French masker speech because Dutch and English share rhythm properties as stress-timed languages that they do not share with French, a syllable-timed language. Because the study did not include manipulation of only speaking rate without f0 manipulations using Dutch and French maskers, any potential effects of speaking rate were overwhelmed by the f0 effects. A future study comparing Dutch and French maskers that only manipulates speaking rate may identify whether any differences in Speaking Rate Match occur.

Because any differences in participant performance between target stimuli within Dutch or French masker babble could be due to a variety of other factors beyond rhythm properties, including sound inventory, future studies may additionally want to compare another stress-timed language with a relatively distinct phoneme inventory to identify whether behavior differs for this language compared to Dutch.

Overall, the present research identified a clear role of f0 in segregating target speech from masker babble. While speaking rate can help in segregating target speech from masker babble, when a more salient cue that is more reliable in cuing the presence of a new talker, such as f0, is available, speaking rate, which is a less reliable cue for speaker identity, no longer seems to matter. When the masker babble is in a language unknown to listeners, such as Dutch or French, masking is less effective compared to when the masker is in a known language to listeners. The present study offers some evidence in support of the target-masker linguistic similarity hypothesis [38], indicating that the greater the similarity between target and masker, the more effective the masker is at masking target speech. Due to f0’s greater ability to cue speaker identity, f0 cues were more effectively used by participants than speaking rate cues, which are typically less effective at signaling speaker identity, when both cues were available. Moreover, significant additive effects of f0 and speaking rate were not found. Instead, f0 effects were more influential than speaking rate effects. Future research should test various F0 and speaking rate differences against each other to more definitively determine whether F0 is always used as the primary cue over speaking rate when both are present or whether, or to what extent, the ranges of differences between speaking rates and F0 values influence cue usage by listeners.

This study is the first of its kind to attempt to identify the role of speaking rate differences in competing speech perception, offering motivation for continuing future research to identify how speaking rate may interact with other acoustic cues in speech segregation.

The present finding that f0 and speaking rate behave differently, likely as a result of their differences in ability to reliably signal the identity of a talker, also brings into question what other acoustic cues to speaker identity listeners may be attending to and how these cues rank against each other in order of importance when trying to identify a target speaker in noisy listening conditions. Future research further defining the identity cues that can be most reliably used by listeners to track a speaker in suboptimal listening conditions can offer insight into how to more effectively aid listeners in communicating in noisy environments.

The present study identified a differential role of f0 versus speaking rate in speech segregation based on the two cues’ ability to reliably signal the identity of a talker. More specifically, the current results suggest that while cues to speaker identity play an important role in speech segregation, listeners use whatever information is available in the speech signal unless a stronger cue is present. Moreover, the finding that listeners process background speech even when they do not know the language indicates that its acoustic characteristics matter. The focus on understanding the cognitive processes underlying speech perception in noisy ecologically valid contexts, such as in degraded conditions with background speech, allows for a better understanding of real-world speech processing.

## Figures and Tables

**Table 1:** Mixed-effects logistic regression analysis with best fit for accuracy scores for the sentence transcription task in Experiment 1 (full model prior to backwards fitting: Accuracy ~ f0 Match * Target f0 + (1 + f0 Match * Target f0|Subject) + (1 + f0 Match * Target f0|Item); best fitting model: Accuracy ~ f0 Match + (1 + f0 Match|Subject) + (1|Item)).

**Fixed Effects**	**Estimate**	**Std. Error**	***t***	***p***
(Intercept)	0.207	0.251	0.826	0.409
f0 Match (mismatch)	0.726	0.212	3.421	<0.001
**Random Effects**	**Group**	**Variance**	**SD**	**Corr**
Intercept	Item	1.46	1.208	−
Intercept	Subject	0.862	0.929	−
f0 Match slope	Subject	3.421	0.321	−0.15

**Table 2:** Mixed-effects logistic regression analysis with best fit for accuracy scores for the sentence transcription task in Experiment 2 (full model prior to backwards fitting: Accuracy ~ Speaking Rate Match * Target Speaking Rate + (1 + Speaking Rate Match * Target Speaking Rate|Subject) + (1 + Speaking Rate Match * Target Speaking Rate| Item); best fitting model: Accuracy ~ Speaking Rate Match + Target Speaking Rate + (1|Subject) + (1|Item)).

**Fixed Effects**	**Estimate**	**Std. Error**	***t***	***p***
(Intercept)	−0.1067	0.307	−0.348	0.728
Speaking Rate Match (mismatch)	0.413	0.194	2.131	0.033
Target Speaking Rate (slow)	0.869	0.412	2.107	0.035
**Random Effects**	**Group**	**Variance**	**SD**	**Corr**
Intercept	Item	1.372	1.171	-
Intercept	Subject	0.663	0.814	-

**Table 3:** Mixed-effects logistic regression analysis with best fit for accuracy scores for the sentence transcription task in Experiment 3 (full model prior to backwards fitting: Accuracy ~ f0 Match * Speaking Rate Match + (1 + f0 Match * Speaking Rate Match| Subject) + (1 + f0 Match * Speaking Rate Match| Item); best fitting model: Accuracy ~ f0 Match + Speaking Rate Match + (1 + Speaking Rate Match| Subject) + (1 + Speaking Rate Match| Item)).

**Fixed Effects**	**Estimate**	**Std. Error**	***t***	***p***
(Intercept)	0.34	0.152	2.241	0.025
f0 Match (mismatch)	0.572	0.143	4.008	<0.001
Speaking Rate Match (mismatch)	0.228	0.144	1.584	0.113
**Random Effects**	**Group**	**Variance**	**SD**	**Corr**
Intercept	Item	1.573	1.254	-
Speaking Rate Match slope	Item	0.536	0.732	−0.32
Intercept	Subject	0.696	0.834	-
Speaking Rate Match slope	Subject	0.051	0.226	0.32

**Table 4:** Mixed-effects logistic regression analysis with best fit for accuracy scores for the sentence transcription task in Experiment 4 (full model prior to backwards fitting: Accuracy ~ f0 Match * Speaking Rate Match + (1 + f0 Match * Speaking Rate Match| Subject) + (1 + f0 Match * Speaking Rate Match| Item); best fitting model: Accuracy ~ f0 Match + (1|Subject) + (1|Item)).

**Fixed Effects**	**Estimate**	**Std. Error**	***t***	***p***
(Intercept)	1.166	0.173	6.728	<0.001
f0 Match (mismatch)	0.400	0.153	2.615	0.009
**Random Effects**	**Group**	**Variance**	**SD**	**Corr**
Intercept	Item	1.705	1.306	-
Intercept	Subject	0.829	0.911	-

**Table 5:** Mixed-effects logistic regression analysis for accuracy scores for the sentence transcription task in Experiment 5 (full model prior to backwards fitting: Accuracy ~ f0 Match * Speaking Rate Match + (1 + f0 Match * Speaking Rate Match| Subject) + (1 + f0 Match * Speaking Rate Match| Item); best fitting model: Accuracy ~ f0 Match + Speaking Rate Match + (1 + Speaking Rate Match + f0 Match|Subject) + (1 + Speaking Rate Match + f0 Match|Item)).

**Fixed Effects**	**Estimate**	**Std. Error**	***t***	***p***	
(Intercept)	0.945	0.19	4.971	<0.001	
f0 Match (mismatch)	0.227	0.167	1.358	0.174	
Speaking Rate Match (mismatch)	0.054	0.17	0.317	0.751	
**Random Effects**	**Group**	**Variance**	**SD**	**Corr**	
Intercept	Item	2.343	1.531	-	-
Speaking Rate Match slope	Item	0.392	0.626	−0.66	-
f0 Match Slope	Item	1.333	1.155	−0.4	0.39
Intercept	Subject	0.623	0.79	-	-
Speaking Rate Match slope	Subject	0.085	0.291	−0.12	-
f0 Match slope	Subject	0.057	0.238	−0.59	0.37

**Table 6:** Mixed-effects logistic regression analysis with best fit for accuracy scores for the sentence transcription task in Experiments 3, 4, and 5 (full model prior to backwards fitting: Accuracy ~ f0 Match * Rate Match * Language + (1 + f0 Match + Speaking Rate Match|Subject) + (1|Item); best fitting model: Accuracy ~ f0 Match + Speaking Rate Match + Language + (1 + f0 Match + Speaking Rate Match|Subject) + (1|Item)).

**Effect**	**Estimate**	**Std. Error**	***t***	***p***	
(Intercept)	1.095	0.134	8.187	<0.001	
f0 Match (mismatch)	0.403	0.088	4.559	<0.001	
Speaking Rate Match (mismatch)	0.132	0.089	1.482	0.138	
Language (English)	−0.628	0.107	−5.846	<0.001	
Language (French)	−0.29	0.111	−2.619	0.009	
**Random Effects**	**Group**	**Variance**	**SD**	**Corr**	
Intercept	Item	1.705	1.306	-	-
Intercept	Subject	0.734	0.857	-	-
f0 Match slope	Subject	0.016	0.128	−0.18	-
Speaking Rate Match slope	Subject	0.027	0.164	0.07	0.07

## Data Availability

The data that support the findings of this study are available at this Open Access Repository: OSF|Speech-in-speech perception (https://osf.io/tfuqh/files/osfstorage).
